# General practitioners’ knowledge, attitudes and experiences of managing behavioural and psychological symptoms of dementia: protocol of a mixed methods systematic review and meta-ethnography

**DOI:** 10.1186/s13643-018-0732-7

**Published:** 2018-04-23

**Authors:** Aisling A. Jennings, Tony Foley, Kieran A. Walsh, Alice Coffey, John P. Browne, Colin P. Bradley

**Affiliations:** 1 Department of General Practice, University College Cork, Cork, Ireland; 2 School of Public Health, University College Cork, Cork, Ireland; 3 Pharmaceutical Care Research Group, School of Pharmacy, University College Cork, Cork, Ireland; 4 Centre for Gerontology and Rehabilitation, School of Medicine, University College Cork, Cork, Ireland; 5 Department of Nursing and Midwifery, University of Limerick, Limerick, Ireland

**Keywords:** Dementia, General practitioners, Behavioural and psychological symptoms of dementia (BPSD), Neuropsychiatric symptoms (NPS), Knowledge and attitudes, Qualitative research, Mixed methods, Systematic review, Protocol, Meta-ethnography

## Abstract

**Background:**

In the context of rising dementia prevalence, the workload of general practitioners (GPs) in dementia care is set to increase. However, there are many aspects of dementia care that GPs find challenging. Behavioural and psychological symptoms of dementia (BPSD) affect the majority of people with dementia and is an aspect of dementia care that GPs find particularly difficult to manage. The aim of this mixed methods systematic review is to undertake a synthesis of qualitative and quantitative studies on GPs’ knowledge, attitudes and experiences of managing BPSD.

**Methods:**

Seven electronic bibliographic databases will be searched from inception to present. All qualitative or quantitative studies that explore the knowledge, attitude or experiences of GPs towards the management of BPSD in community and/or residential settings will be eligible for inclusion. A meta-ethnography will be conducted to synthesise included studies. Primary outcome measures will include GPs’ experiences of managing BPSD, GPs’ knowledge of BPSD and their attitude to different approaches to the management of BPSD, in particular their attitude to non-pharmacological approaches. All included papers will be independently assessed for methodological validity by two reviewers using the following tools: the Joanna Briggs Institute checklist for qualitative research, the Effective Public Health Practice Project (EPHPP) tool for intervention studies and the National Institute of Health (NIH) quality assessment tool for observational and analytical cross-sectional studies. As there is no agreed quality assessment tool for descriptive cross-sectional studies, an original tool will be developed. Two independent reviewers will apply the Confidence in the Evidence from Reviews of Qualitative Research (CERQual) tool to the review findings. The results will be reported in line with the Enhancing Transparency in Reporting the Synthesis of Qualitative Research (ENTREQ) statement.

**Discussion:**

This study will be the first systematic review that synthesises the existing literature of GPs’ knowledge, attitudes and experiences of managing BPSD in community and residential care. This review will improve our understanding of GPs’ perspectives on the management of BPSD, and the results will be used to inform the development of an intervention to improve the management of BPSD in general practice.

**Systematic review registration:**

PROSPERO CRD42017054916.

**Electronic supplementary material:**

The online version of this article (10.1186/s13643-018-0732-7) contains supplementary material, which is available to authorized users.

## Background

General practitioners play a pivotal role in the care of a person with dementia and their families [1]. It is estimated that there are currently 47 million people living with dementia worldwide, and this figure is predicted to triple by 2050 [2]. In the context of rising dementia prevalence [3], the dementia workload of general practitioners (GPs) is set to increase further. National dementia strategies have been developed internationally to respond to the challenge posed by increasing dementia prevalence and have emphasised the central role of GPs in successful implementation [4–6]. GPs find many aspects of dementia care, such as diagnosis disclosure and co-ordinating support services, to be challenging [7]. However, the one area that consistently emerges as a particularly challenging aspect of dementia care for GPs internationally is the management of behavioural and psychological symptoms of dementia (BPSD) [7–11].

BPSD encompasses a wide range of symptoms and behaviours that affect people with dementia. BPSD includes behaviours such as aggression, wandering, sexual disinhibition and agitation and symptoms such as anxiety, depression and delusions. These symptoms and behaviours often overlap and occur together rather than occurring as isolated symptoms [12]. The majority of people with dementia will experience BPSD [13]. Estimates of BPSD prevalence vary [14, 15], and we know the presence of BPSD can be influenced by several factors including dementia severity [16]; however, some studies estimate that up to 80% of people with dementia experience at least one symptom of BPSD at some stage in their illness [15]. The presence of BPSD results in increased rates of admission to long-term care facilities [17, 18] and longer in-patient hospital stays [19]. The development of BPSD is also associated with a worse prognosis for the patient and a more rapid rate of illness progression [20]. From a carer perspective, BPSD is a major contributor to stress and depression, even more significant than cognitive decline [21]. For physicians, the assessment of BPSD is complex, and effective treatment options are limited [22]. Antipsychotics are associated with serious adverse effects including stroke [23–25] and are not recommended unless there is a serious risk to self or others [26]; however, credible pharmacological alternatives remain scarce [27]. There is agreement that in most cases, non-pharmacological interventions should be used first line [28]; however, effective non-pharmacologic strategies for BPSD have not been translated into real-world clinical practice [29] and are not viewed by many GPs as being credible options [30]. Many people with dementia experiencing BPSD may be under active care with secondary care services; however, GPs describe difficulty accessing advice from these services [30, 31].

If GPs are to play the pivotal role described in the various national strategies, then interventions will be needed to support GPs in their management of BPSD; however, we are unaware of any such interventions. An important first step in intervention design is to establish a thorough understanding of existing behaviour [32, 33]. To date, no qualitative or quantitative synthesis has been performed on studies which focused on GPs’ perspectives on the management of BPSD in community and residential care settings. Primary quantitative studies performed to date [30, 34] have been conducted in different contexts and at different times in the evolution of the management of BPSD. Likewise, qualitative studies in this area [35, 36] were conducted in different healthcare systems and took different approaches to the evidence. Exploring these contextual differences will improve the depth of our overall understanding of the research question. In order to effectively address our research aim, we will include both relevant quantitative and qualitative studies, as a review which “focuses exclusively on one form of evidence presents only half the picture and thus will have limited applicability” [37].

The aim of this mixed methods systematic review is to develop a synthesis of qualitative and quantitative studies on GPs’ knowledge, attitudes and experiences of managing BPSD in order to develop a conceptual understanding of the perspective of GPs on the management of BPSD. The results of this systematic review will subsequently inform the development of a future behavioural change intervention.

## Methods

This review protocol was developed using the Preferred Reporting Items for Systematic Reviews and Meta-Analyses Protocol (PRISMA-P) [38] (see Additional file 1). The systematic review was registered with the International Prospective Register of Systematic Reviews (PROSPERO) on the 11th of January 2017 and was last updated on the 25th of July 2017 (registration number 42017054916).

### Methodological framework

**Fig. 1:**
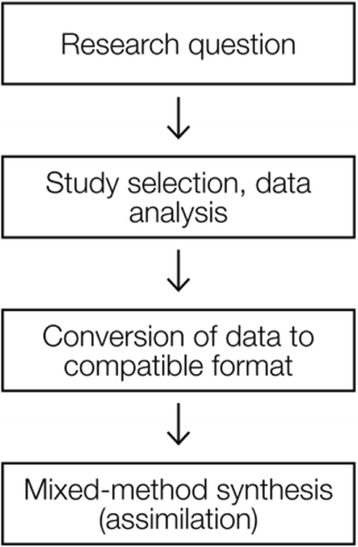
The integrated approach to mixed methods systematic review (*adapted from the JBI Reviewers’ Manual: Mixed Methods Systematic Reviews*)

This mixed methods systematic review will take an integrated approach to synthesis as described by Sandelowski et al. [37, 39]. The integrated approach involves assimilating study findings into each other as opposed to segregating the qualitative and qualitative synthesis (see Fig. 1) [39, 40]. The assimilation approach is particularly appropriate when findings are viewed as confirming each other or converging in the same direction [40]. The integrated approach will involve transforming quantitative data, usually obtained from GPs’ responses to standardised questionnaires, into qualitative form so that it can be combined with data from qualitative studies and subjected to qualitative analysis. This approach has been used effectively in previous mixed methods systematic reviews of similar research questions [41, 42]. Once the data is in qualitative form, our approach to qualitative synthesis will follow the seven-step model of meta-ethnography as described by Noblit and Hare [43] (see Table 1). Meta-ethnography is explicit when describing the act of ‘translation’ where terms and concepts which have resonance are enveloped into ‘high-order constructs’ [44] and goes beyond merely describing or summarising the data allowing an original interpretation of the topic under review.

**Table 1:** Seven steps of Noblit and Hare’s meta-ethnography

1. Getting started 2. Deciding what is relevant to the initial interest 3. Reading the studies 4. Determining how the studies are related 5. Translating the studies into one another 6. Synthesising translations 7. Expressing the synthesis	

Syntheses of qualitative data have been criticised as being mechanistic. Indeed, there is the risk with meta-ethnography that the richness or integrity of the original work will be lost [45], a concern that by overly deconstructing the original qualitative work, the researcher attempts to “sum up a poem” [46]. However, when conducted rigorously, a synthesis of qualitative studies leads to a more substantive interpretation of the research phenomenon than is available from a single study [47]. Rather than attempting to totalise concepts, a synthesis of qualitative literature aims to offer fresh new insights into the phenomenon of interest [48]. In order to achieve a deeper understanding of the shared meanings of the area under review, it is essential that rigour is applied to each stage of the review process. In this review, all efforts will be made to retain the content and context of the original studies throughout the data extraction and analysis. Each stage of the review process will involve at least two authors working independently. At every stage, a third author, experienced in performing meta-ethnographic synthesis, will be available for consultation.

We will report our results in line with the Enhancing Transparency in Reporting the Synthesis of Qualitative Research (ENTREQ) statement [49], and we will express our search strategy results using the Preferred Reporting Items for Systematic Reviews and Meta-Analyses (PRISMA) flow diagram [50].

### Eligibility criteria

Qualitative or quantitative studies that explore the knowledge, attitude or experiences of GPs towards the management of BPSD in community and/or residential settings will be eligible for inclusion. All study designs will be included. Qualitative studies that focus more generally on GPs’ perspectives on dementia management will be included only if there is a specific reference to BPSD in the results. Quantitative studies that focus on the knowledge and attitude of GPs to other aspects of dementia care will only be included if there is a specific reference to BPSD in the results. Randomised control trials and other intervention studies will be included in the final review if they identify the knowledge base or attitude of GPs towards BPSD during the study. Opinion pieces and non-peer-reviewed articles will be excluded. Studies not written in the English language will be excluded. This is due to resource limitations which prevent employment of formal translation services. However, if eligible non-English language studies are identified, we will attempt to contact the study authors to see if there are any English translations available. A list of possibly relevant titles in other languages will be provided as an additional file. Studies that do not describe in detail the knowledge and attitudes of GPs in relation to BPSD will be excluded. Studies that report on the perspective of non-GP healthcare professionals to BPSD in addition to GPs will be included so long as the views of GPs are represented or analysed separately (see Table 2 for the eligibility criteria).

**Table 2:** Eligibility criteria for studies in the systematic review

Inclusion criteria	Exclusion criteria
- Studies that explore the knowledge, attitude or experiences of GPs in the management of BPSD in community and/or residential settings - Qualitative or quantitative study design - Studies must include GPs	- Studies that do not describe in detail the knowledge and attitudes of general practitioners in relation to BPSD - Non-English language studies - Studies reporting the perspective of non-GP healthcare professionals where the views of GPs are not represented or analysed separately - Studies reporting on GPs’ perspectives on managing another aspect of dementia without any reference to the management of BPSD - Opinion pieces and non-peer reviewed articles

### Information sources and search strategy

We will search the following seven electronic bibliographic databases from inception to present with no date limits: MEDLINE (Ovid) 1946–present, EMBASE (Elsevier), CINAHL, PsycINFO, Academic Search Complete, SocIndex and Social Science Full Text. The search strategy has been developed using database-specific search terms with input from the review team and a health services librarian with expertise in systematic review searching. The MEDLINE search strategy is included in Additional file 2. Other search methods utilised will include the following: hand-searching key journals and conference proceedings, forward citation searching of eligible studies and searching reference lists of included studies.

### Data management

A flow diagram using PRISMA guidelines will be used to report the selection process and all results. The results of our search will be exported to Covidence (www.covidence.org). Duplicates will be identified and removed. Covidence will then be used to manage citations and perform title and abstract screening.

### Study selection

At the first stage, duplicates and clearly irrelevant studies (for example pre-clinical studies) will be removed. In the next stage, abstract screening will be conducted. To manage the workload that may result from a large number of citations, four reviewers (AJ, TF, AC, CB) will form three paired teams: AJ and TF, AJ and AC, AJ and CB. The search results will be randomly divided into three groups and assigned to a paired team. The two reviewers in each paired team will independently screen each study abstract and assess the study’s suitability for inclusion based on pre-determined inclusion and exclusion criteria. Conflicts will be resolved through discussion, and where necessary, a third reviewer, selected from a different paired team, will act as adjudicator. Subsequently, all potentially eligible studies included in full-text screening will be assigned to a paired team for eligibility assessment. Any conflicts regarding the eligibility of a study at full-text screening will be resolved through discussion between the two members of the paired team. Where consensus is not reached through discussion, a third reviewer, selected from a different paired team, will adjudicate and make the final decision regarding inclusion. All studies that are excluded after full-text screening will be displayed, with their reason for exclusion, as an additional file in a table form.

### Data extraction, analysis and synthesis

We will follow the meta-ethnographic approach as described by Nobilt and Hare when extracting, analysing and synthesising the data. This stage of the review process maps to steps 3–7 of the meta-ethnographic approach [see Table 1].

#### Data extraction

The data extraction and analysis stage will involve four of the reviewers (AJ, TF, KW, CB). All four reviewers will independently read and re-read all the eligible studies in chronological order focusing initially on the content and context (step 3 of meta-ethnography approach). Data concerning participant characteristics, aims, setting and methods will be extracted independently by two reviewers (AJ, TF) and displayed in tabular form. Data extraction will be facilitated by the use of standardised data extraction tables. The data extraction forms will be pilot tested by the reviewers on the first two included studies to ensure consistency and reliability between reviewers. A third reviewer (KW) will oversee the data extraction process and will be available for consultation. If necessary, we will contact the study authors to resolve any uncertainties. Table 3 shows data categories that will be extracted from all the included studies.

**Table 3:** Data extraction categories

1. Author 2. Date 3. Country 4. Study objectives 5. Study design 6. Analysis 7. Participant characteristics 8. Setting	

#### Data analysis and synthesis

**Fig. 2:**
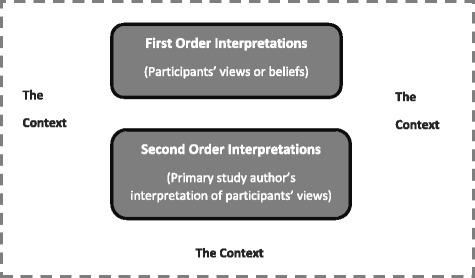
First- and second-order interpretations

The lead author (AJ) will open code all the included studies focusing specifically on the first and second-order interpretations (Fig. 2). First-order interpretations refer to the participants’ views as they are reported in the results section of the included study. In the qualitative studies, the first-order interpretations will focus on attitudes and experiences of GPs. In the quantitative studies, the first-order interpretations will involve creating a text file that describes participants’ responses to questionnaire items. In the studies that include other healthcare professionals, the study findings, where possible, will be restricted to the views of GPs. Second-order interpretations refer to the original study author’s interpretation of the participants’ views usually found in the discussion section. In the qualitative papers, author-derived themes, conclusions, interpretations and recommendations will form the basis of the second-order interpretations. In the quantitative studies, the second-order interpretations will be derived from the results, recommendations and conclusions. The data will be extracted verbatim for all the included studies to ensure no valuable detail is lost. All efforts will be made to retain the context of the findings from both the qualitative and the quantitative studies during data extraction [51]. At this point, the data collected from quantitative and from qualitative studies will be no longer distinguishable in terms of study design, enabling the synthesis of all the data in qualitative form.

We acknowledge that performing the second-order interpretations can be challenging as the value of second-order constructs lies to an extent in the richness and depth of the analysis performed by the original authors [52]. To ensure credibility and dependability of coding, a second reviewer (KW) will code a random selection of studies. Conceptual groupings for each study will be created and illustrated with the development of conceptual mind maps. The two reviewers involved (AJ, KW) will meet regularly to discuss the differences in interpretation of the studies. A third reviewer (CB) will oversee the data analysis process and will be available for consultation. Finally, all four members of the data extraction and analysis team (AJ, KW, CB, TF) will meet to discuss the key concepts emerging from the analysis of the included studies. The software package NVivo version 11 will be used to facilitate data analysis and synthesis.

Step 4 of the meta-ethnographic approach involves *determining how studies are related* to each other. To facilitate this step, a table will be developed to display the identified concepts and themes across all the studies. Relationships between the conceptual groups and themes will be organised and illustrated by the use of conceptual maps. Step 5 of the meta-ethnography involves *translating the studies into one another*. To examine the contribution of each study to a key concept, the review team will compare the themes and concepts from paper 1 with paper 2 and the synthesis of these two papers with paper 3 and so on. This process will be conducted in chronological order starting with the earliest study [52]. A chronological approach is appropriate as the included studies are likely to range over multiple decades, during which time significant changes in the management of BPSD occurred. Within the key concepts, attention will be paid to deviant cases. Two authors (AJ, KW) will perform reciprocal and refutational analyses to summarise shared themes across the studies. We will attempt at all times to consider the influence of context to the study finding; however, we acknowledge that this may be difficult as previous meta-ethnographies have reported on the challenges of retaining the context of the primary studies when contextual information is often poorly reported [52]. Step 6 will involve *synthesising the translations* in each key concept to iteratively develop third-order interpretations. A synthesis of the first- and second-order interpretations and the third-order interpretations constructs a new model or theory about a problem. The synthesis team (all authors) will link the third-order interpretations into a ‘line of argument’ which will represent the overarching perspective of GPs towards BPSD. The final step in the meta-ethnography approach involves *expressing the results of the synthesis*. For this step, we will use tables, figures and text.

#### Assessment of confidence in the study findings

Two independent reviewers (AJ, KW) will apply the Confidence in the Evidence from Reviews of Qualitative Research (CERQual) tool to the review findings (i.e. third-order interpretations) as conducted in a recent meta-ethnography [53]. The CERQual approach provides a transparent method of assessing the confidence of findings of systematic reviews of qualitative research [54]. There are four key components to the CERQual approach: (i) methodological limitations of the qualitative studies contributing to a review finding, (ii) the relevance to the review question of the studies contributing to a review finding, (iii) the coherence of the review finding and (iv) the adequacy of data supporting a review finding. Judgements relating to each CERQual component will be summarised in table form. Each review finding will be rated and given an assessment of confidence as high, moderate, low or very low. We will assign high confidence if it is highly likely, moderate confidence if it is likely, low confidence if it is possible and very low confidence if it is unclear if the review finding is a reasonable representation of the phenomenon of interest [54].

### Outcomes

Primary outcome measures will include GPs’ experiences of managing BPSD, especially their confidence in this field. GPs’ knowledge of strategies to manage BPSD and their attitude to different approaches to the management of BPSD, in particular the role of non-pharmacological approaches, will also be included. Additionally, we will seek to identify data on GPs’ needs with respect to skill levels and competencies in this field.

### Quality assessment

All included papers will be independently assessed by two reviewers (AJ, JB) for methodological validity. Agreement on the quality assessment will be measured using Cohen’s Kappa, and in consideration of previous literature in this area, values greater or equal to 0.6 will be considered an acceptable level of agreement [55]. Disagreements will be resolved by discussion between the two reviewers. Given the large number of study designs that will potentially be included in the study, a number of quality assessment tools will be required.

The quality assessment tools that will be used to assess the quality of the quantitative studies have been agreed through consultation with the systematic review team. The Effective Public Health Practice Project (EPHPP) tool will be used for intervention studies [56]. The National Institute of Health (NIH) quality assessment tool for observational and analytical cross-sectional studies will be used where appropriate [57]. Since there is no agreed quality assessment tool for assessing the quality of descriptive cross-sectional studies, a new original tool will be developed by two of the reviewers (AJ, JB) that will be based on other original tools developed for a similar purpose [42, 58]. This new tool will also consider recommendations on how survey questionnaires should be designed [59].

There are a number of quality appraisal tools available for assessing the quality of qualitative studies [60]. However, it is recognised that critical appraisal instruments for qualitative research differ in the criteria they apply to a critical appraisal process [61]. On examining potential quality assessment tools, it is clear that many of the existing appraisal instruments for qualitative research use quite broad criteria that often reflect the quality of the *reporting* of the research rather than addressing the core quality issues inherent to qualitative research, such as issues relating to the credibility, dependability, confirmability and transferability of the research. Qualitative studies may rate as “low quality” when assessed as a result of methodological flaws, a poorly designed quality assessment tool or simply because of lack of reporting, which can often be a consequence of meeting tight word count deadlines for journals. However, these studies may still generate novel concepts and insights [62]. As Dixon-Woods observes, some of the most important qualities of qualitative research can be the hardest to measure [63]. Appraisal tools, generally, focus on the methodological strength of the paper rather than its conceptual strength [64]. However, a qualitative study that has clearly reported its methods may not generate rich interpretation of the phenomenon of interest. Likewise, a qualitative study that appears to have face validity and offers rich, insightful interpretations might not necessarily do well on quality assessment [52]. This then leads to questions on how the quality of qualitative can be legitimately judged or indeed whether it should be judged at all [65].

We have chosen to assess the quality of the qualitative studies. However, quality appraisal will not be used to exclude qualitative or quantitative studies that otherwise meet the inclusion criteria. The CERQual assessment requires an evaluation of the methodological limitations of each of the studies that supports each third-order interpretation [54]. Therefore, the quality assessment given to the studies will influence the confidence rating we can give to each review finding. A poor quality assessment will not, on its own, alter the confidence assessment, but the results of quality assessment will be considered as part of a wider assessment of the confidence we have in our review findings.

Following a process of consultation and discussion between the members of the review team, the Joanna Briggs Institute Critical Appraisal Checklist for Qualitative Research was chosen as the quality assessment tool that will be used to assess the qualitative studies [66]. This particular quality assessment tool was chosen as it was found to focus on the quality of the study design rather than just the reporting rigour. Additionally, this quality assessment tool is specifically designed for use in systematic reviews.

## Discussion

This study will be the first systematic review that synthesises the existing literature of GPs’ knowledge, attitudes and experiences of managing BPSD in community and residential care. This review will contribute to improved understanding of GPs’ perspectives on the management of BPSD. We know that BPSD is a challenging area of dementia care for GPs [7, 8]; however, this mixed methods synthesis of all the available quantitative and qualitative research in this field will offer fresh insights and interpretations into *why* this is a challenging area for GPs. The findings of this review can then be used to inform the development of interventions to improve the management of BPSD in primary care. We believe this review will expose gaps in the literature, gaps that should be the focus of future research. Additionally, this review will be valuable to policymakers and health care providers who are attempting to implement national dementia strategies, as many of these strategies hinge upon general practitioners taking on an increasing amount of dementia care. In order to effectively implement these strategies, the current barriers and facilitators of managing this particularly challenging aspect of dementia in primary care need to be identified and addressed. The use of CERQual will provide policymakers with a transparent method for assessing the confidence of the review findings.

### Strengths and limitations

This systematic review is being conducted as part of a wider national project which is one of the implementation work streams of the Irish National Dementia Strategy [5]. Due to time constraints associated with the wider project, this review will not include a search of the grey literature. However, since our search of the electronic databases will be extensive, we feel that the grey literature is unlikely to result in any additional eligible peer-reviewed study.

Existing validated approaches for synthesising quantitative and qualitative data for mixed methods systematic reviews will be followed [39, 40]; however, we recognise that the potential heterogeneity of the evidence may make this synthesis challenging. The benefit of using a mixed methods approach here is that it will enable us to integrate the quantitative assessments of GPs' knowledge of and attitudes towards BPSD with a more qualitative understanding of GPs' experiences of BPSD. Combining these two sources of data into a systematic review will enhance the review’s utility and impact. The development of a new original tool to assess the quality of descriptive cross-sectional studies will be a strength of this review. The tool will be useful for researchers undertaking similar mixed methods systematic reviews. Finally, the application of the CERQual tool to our review findings will provide a validated summary of the confidence we have in the study findings.

## Additional files

Additional file 1: PRISMA-P Checklist. (DOCX 19 kb)

Additional file 2: The MEDLINE, Ovid search strategy. (DOCX 18 kb)

## Abbreviations

BPSD: Behavioural and psychological symptoms of dementia
CERQual: Confidence in the Evidence from Reviews of Qualitative Research
GPs: General practitioners
