# Supplementary material for: General practitioners’ knowledge, attitudes and experiences of managing behavioural and psychological symptoms of dementia: protocol of a mixed methods systematic review and meta-ethnography
Source: Syst Rev. 2018 Apr 23;7:62. doi: 10.1186/s13643-018-0732-7 (PMC5913890; doi:10.1186/s13643-018-0732-7)
Supplement: Supplementary file 2 — The MEDLINE, Ovid search strategy. (DOCX 18 kb) [file 13643_2018_732_MOESM2_ESM.docx]

**Additional file 2: The Medline, Ovid search strategy**

|  | **Primary Care Physicians** | **Dementia** | **BPSD** |
| --- | --- | --- | --- |
| **MeSH Terms/ Subheadings** | Exp Primary Health Care/  Exp General Practice  Family Practice/  Exp General Practitioners/  Exp Physicians, Family/  Exp Physicians, Primary Care/ | Exp Dementia/  Exp Alzheimer Disease/ | Exp Antipsychotic Agents/  Exp Anxiety/  Exp Aggression/  Exp Wandering behavior/  Exp Sleep Disorders/  Exp Apathy/  Exp Irritable Mood/  Exp Psychotic Disorders/  Exp Depression/ |
| **Text Words** | family medicine.ti,ab | dementia.ti,ab. alzheimer*.ti,ab.  (cognitive adj (impairment or decline)).ti,ab | Behavio?ral and psychological symptom*ti,ab  BPSD.ti,ab  Challenging behavio?r*ti,ab  Responsive behavio?r*.ti,ab  Neuropsychiatric symptom*.ti,ab  Non-cognitive symptom*.ti,ab  Noncognitive symptom*.ti,ab  Psychological symptom*.ti,ab  Psychiatric symptom*.ti,ab  Difficult behav*.ti,ab  Disruptive behav*.ti,ab  Behavio?ral symptom*.ti,ab  (agitated or agitation).ti,ab  (depressed or depression).ti,ab  (anxiety or anxious).ti,ab (aggressive* behav*).ti,ab |

How the search terms will be combined with Boolean logic for the Medline, Ovid search

1. Exp Primary Health Care/
2. Exp General Practice
3. Family Practice/
4. Exp General Practitioners/
5. Exp Physicians, Family/
6. Exp Physicians, Primary Care/
7. family medicine.ti,ab
8. 1 OR 2 OR 3 OR 4 OR 5 OR 6 OR 7
9. Exp Dementia/
10. Exp Alzheimer Disease/
11. dementia.ti,ab. alzheimer*.ti,ab.
12. (cognitive adj (impairment or decline)).ti,ab
13. 9 OR 10 OR 11 OR 12
14. Exp Antipsychotic Agents/
15. Exp Anxiety/
16. Exp Aggression/
17. Exp Wandering behavior/
18. Exp Sleep Disorders/
19. Exp Apathy/
20. Exp Irritable Mood/
21. Exp Psychotic Disorders/
22. Exp Depression/
23. Behavio?ral and psychological symptom*ti,ab
24. BPSD.ti,ab
25. Challenging behavio?r*ti,ab
26. Responsive behavio?r*.ti,ab
27. Neuropsychiatric symptom*.ti,ab
28. Non-cognitive symptom*.ti,ab
29. Noncognitive symptom*.ti,ab
30. Psychological symptom*.ti,ab
31. Psychiatric symptom*.ti,ab
32. Difficult behav*.ti,ab
33. Disruptive behav*.ti,ab
34. Behavio?ral symptom*.ti,ab
35. (agitated or agitation).ti,ab
36. (depressed or depression).ti,ab
37. (anxiety or anxious).ti,ab (aggressive* behav*).ti,ab
38. 14 OR 15 OR 16 OR 17 OR 18 OR 19 OR 20 OR 21 OR 22 OR 23 OR 24 OR 25 OR 26 OR 27 OR 28 OR 29 OR 30 OR 31 OR 32 OR 33 OR 34 OR 35 OR 36 OR 37
39. 8 AND 13 AND 38
